# Report of Committee on Public Health of the Medical Society of the County of Erie

**Published:** 1912-02

**Authors:** 


### SOCIETY PROCEEDINGS

### Report of Committee on Public Health of the Medical Society of the County of Erie.

To the 90th annual meeting of the Medical Society of the County of Erie, 1911.

Mr. President—Your Committee on Public Health would respectfully report—The incidents of the year of particular value to those concerned in matters of preventive medicine have been varied, interesting, and important. To some of these we would invite your consideration. The report of the committee for 1910 called attention to our method of building houses for our public schools. That in some *seventeen* instances cited the specifications in regard to the ventilation of these buildings had not been complied with but on the contrary had been violated in each instance, in that there was no record of the tests of the actual performance of the ventilating apparatus as demanded by the *plain provisions of the contracts,* and also as imperatively demanded by the interests of the teachers and the children of our schools.

Your committee is pleased to be able to report that this matter has been given consideration by the heads of the several departments of our city government chiefly concerned, with the assurance that the evils mentioned will be corrected and in the future that the provisions of contracts will be followed with exactness. We think this is a step in progress in a matter of prime importance, and advise the society to continue its interests in the question of the ventilation of school buildings. The matter is not only one of singular importance, one concerning which there are conflicting opinions of men more or less well qualified to express opinions, but also a subject which is at this time receiving unusual attention of medical men in various parts of the world. As last year, so now we are of the decided opinion that there are many reasons why our teachers and children should have the advantage of the best ventilation to be procured and there are no sufficient reasons in any case, why they should not at least have as good ventilation as the several contracts plainly describe and for which payment has been made.

For many years America has been noted as the favorite soil for the growth of every possible variety of medical vagary. Eclecticism, Homeopathy, Quimby-Eddyism, Osteopathy, and Naturopathy are some of the many instances of such medical cults. Theoretically these systems of medicine are for the purpose of exploiting the alleged superiority of a given method of treatment of diseases, but practically they frequently furnish the material for opposing some of the provisions of preventive medicine, as expressed in state laws or municipal health ordinances. *During the present year* these camp followers of the army of medicine lead by a member of our Board of Aldermen have made and are now pressing an attack upon the very foundation of state medicine, the elemental principle that the state has the right to protect its people by preventive measures from the ravages, the morbidity, and the mortality of the communicable, the preventable diseases as the same is expressed in the laws and in the practices of every civilized state in the world, by provisions for vaccination and revaccination to prevent small-pox.

Agitation of this antivaccination scheme had been in process of development in this vicinity for many years; it assumed definite proportions on June 20, 1911, when Alderman Fisher introduced a resolution repealing the provisions of the health ordinances regarding the vaccination of pupils of our public schools. Numerous hearings before the committee of Aldermen followed at which your committee were present and participated. These official hearings were supplemented by more arguments before various bodies of teachers, parents, mothers and business men. In order to be prepared for these occasions your committee by the advice of the Council procured a series of lantern slides illustrative of the subject—these slides are likely to become popular; their exhibition is instructive and commands attention.

Regarding this antivaccination campaign we have the distinct conviction that the enemy is well organized and generously financed; the leaders and speakers hate state medicine with a vicious hatred that makes civil and parliamentary speaking or debate well nigh impossible; to meet this attack requires a campaign of education and in the seats of the pupils to be taught will be found many members of the medical profession as that profession is broadly known to the State; in this campaign of education the county medical societies must take a prominent part. As a further step in this important matter, in the firm conviction that efficient vaccination and revaccination is a safe and reliable preventive of small-pox; also that investigation will establish here and now as has been universally observed in other times and other countries that the prevalence and severity of small-pox is in inverse proportion to the efficiency of vaccination and revaccination of the time and people, and also keeping clearly in mind the important fact that in a given people at a given time, the vaccination of that people can never be of higher efficiency than the general standard of competency of preparedness of the whole medical profession of the place and time, the following is submitted for your consideration:

Whereas, there were in America during the last reported decade, 297,288 cases of small-pox with 6,632 deaths and

Whereas, the morbidity and the mortality of small-pox in America are rapidly increasing and

Whereas, our country is the field of an active, aggressive and extensive propaganda of antivaccinationism and

Whereas, education in the principles and importance of preventive measures is the only means of preserving our people from the dangers of the aforesaid propaganda and the consequent presence and increase of small-pox, therefore

*Resolved,* That the Medical Society of the County of Erie hereby requests those in authority, City, State and Nation, to take suitable action to provide that hereafter in reporting cases or deaths from small-pox, mention and record shall be made of the facts and times and efficiency of vaccination and revaccination in every case.

*Resolved,* That copies of this appeal be sent under the seal of this society to the Health Commissioner of Buffalo, to the Health Commissioner of the State of New York, to the Medical Society of the State of New York and to the Public Health and Marine Hospital Service of the United States.

Buffalo for many years, in fact ever since our vital statistics have been systematically recorded has suffered from a scandalously high death rate from typhoid fever. In the sixties, and seventies, and eighties, when many of our people drank from wells, the rate was much higher than since the present water supply from the Niagara River. The present death rate from typhoid fever is more than *five times that of many of the large cities of Europe,* and sanitarians are quite in accord that this blighting death rate is due to the infection constantly found in our drinking water, the source of this infection being our *untreated sewage which we discharge into the Niagara River above any of our intakes*—our new intake at the Emerald channel being more exposed to such sewage than the older one farther down the river. A moments inspection of our sewer systems will make this plain. The sewers of Buffalo divide themselves into two systems: First, the sewers of Hertel Avenue, of Bird Avenue and of Swan Street, the sum of their diameters being 21 feet 6 inches, these by separate openings, discharge their sewage into the Niagara River below the intake of our present pumping station.

Second, the sewers discharging into the Buffalo River and its tributaries and these sewers have a diameter of 64 feet 9 inches. Should we estimate the volumes of sewage of these two systems by their several diameters, it would appear that those infecting the Buffalo River and Harbor carry three times the amount of sewage as those which discharge their infection below our water supply intakes.

We are aware that plausible and popular arguments have been made and probably will be made again to show that the currents of our region in lake and river are such as to provide complete protection to both our old and new intakes.

Concerning such arguments we would observe, that we have listened to them for years, but have never been convinced that they show more than the fact, that at times the currents of the lake and the river are favorable to the protection of our intakes from the infection of our sewage. That this protection is present only when the currents of lake and river are determined by shore lines and gravity without the disturbing influence of winds. However, we should not forget that in this locality winds blow and sometimes, nay frequently rudely, boisterously, fiercely. That the prevailing direction of such winds are from the west, or, from the east; that such winds drive out the infected waters of our harbor through the openings of the breakwaters into the immediate vicinity of our intakes, new and old. That under the influence of such winds the water levels of our harbor rises or falls many feet in height—and further, that from the well-known habits of fluids under pressure when in a lake or harbor from the action of wind a superficial current of water is moving with the wind, there is always a deeper current moving in the opposite direction; and the direction, the velocity and the volume of the one, determines the direction, the velocity and the volume of the other. Thus it will be seen that high winds from the west, or, from the east are capable by superficial or deep currents of contaminating the neighborhood of our intakes, new and old, with the infected waters of our sewage laden Buffalo River and Harbor. That such infection does in fact frequently take place is amply demonstrated by the bacterial findings of our water examinations, by the frequent warnings from our Health office to boil the water—and from our persistent high death rate from typhoid fever.

In the mention of our sewer system we observed that a number of our newer and larger sewers discharged their untreated sewage into the Niagara River below our old intake—this fact is of enormous significance to the neighboring cites, Niagara Falls, Lockport and the Tonawandas. We have had the honesty and the courage to characterize the death rate from typhoid fever of Buffalo as scandalously high; we are ashamed to be obliged to admit that the same for the cities just mentioned is much higher, and the prime cause of the disease is the untreated sewage of Buffalo. The intelligent sanitary consideration of this question will only come when the cities of the Niagara frontier are conceived as one people, one in interest, one in health problems, one in sewage disposal, one in water supply. Buffalo is poisoning her own people, and year by year is giving them more than five times as much typhoid fever as have the cities of Berlin, Hamburg and Munich. Buffalo sewage is fouling the water supply of the Tonawandas with the result that our unfortunate dwellers in those cities have more than ten times the typhoid fever as the foreign cities before mentioned, and in a like manner by the same means Buffalo is infecting the watter supply of Niagara Falls, and in consequence the unfortunate inhabitants of that world famed city of resort have more than thirty times the typhoid fever of those cities of Europe, and yet we of America are wont to boast that we are a practical people, an intelligent people and that we believe and know that in disease, prevention is better and cheaper than cure.

The indifference, the apathy, the ignorance of Americans as to problems of public health is appalling in degree, and most discouraging and disheartening in effect. A dam at Johnstown or Austin breaks as such incompetently constructed structures are wont to break, and for a few days we seem to appreciate the importance of competent advice in such buildings. The roof of a pump house falls as roofs so built are liable to fall, and in falling kills eight of our people, and for a short time we seem to be aroused to some intelligent appreciation of the importance of the work of the builder. And yet Buffalo in 1910, lost by death from the more common preventable diseases more than 143 times as many citizens as were killed when the pump house roof fell, and to this enormous unnecessary killing the usual experience of the years, we are apparently indifferent.

Verily, government of the people, for the people, by the people has not yet achieved perfection. Verily, the work of medical men and of medical societies in educating the American people to a right evaluation of the practical principles of preventive medicine is not a light and trifling task but is a task demanding constant and continuous efforts without cessation or discouragement, even when results are seemingly disappointing. We must fight it out on this line if it takes all summer. Some of our folks occasionally complain of the slow growth of our city. Possibly we may more of us one day appreciate the fact, that a low sick and death rate makes for the financial and commercial prosperity and growth of a city. Possibly we may one day come to see that when we would show to all that "Buffalo means business," we will so care for our sewage, or filter our city water, that it will be no longer necessary to save our citizens from the impending danger of the preventable sickness and death by typhoid fever, by the oft repeated admonition "Boil the Water."

In conclusion we would report with sincere pleasure the considerate courtesy we have received from the heads of the several departments concerned in our work. Our official duty compelled the severe criticism of methods and results, but such criticism has never in corresondence or in inerviews provoked anything but patient kindness and courtesy.

We would also mention the literature consulted in the preparation of this report—"The Reports of the Department of Public Works of Buffalo, N. Y., 1906 to 1910." "Reports of the President's Homes Commission," "Small Pox in the United States, by John W. Trask, Assistant Surgeon General United States Public Health and Marine Hospital Service." "Sewage Pollution of Interstate and International Waters, by Allan J. McLaughlin, Hygienic Laboratory Bulletin No. 77." To these official publications we are greatly indebted, and the obligation we gratefully acknowledge. Respectfully submitted

EDWARD CLARK,
P. W. VAN PEYMA,
HENRY REED HOPKINS,
*Chairman.*

*Committee on Public Health of the Medical Society of the County of Erie, December 18, 1911.*
433 FRANKLIN STREET.

---

THE Medical Society of the County of Cattaraugus, met January 2, at Salamanca, devoting special attention to the matter of vaccination. Officers were elected as follows: President, H. W. Livermore, Gowanda; Vice-President, Benjamin Van Campen of Olean; Secretary-Treasurer, Herman W. Johnson, Gowanda.

---

THE Elmira Academy of Medicine, on January 3, enjoyed a paper on Deafness by Dr. G. M. Case and one on Uterine Cancer by Dr. A. H. Baker.

---

THE Buffalo Academy of Medicine announces the following programs for February. The meetings are held at 8.30 in the Public Library Building and visiting physicians are welcome.

February 6. Surgical Aspects of Certain Organs of Internal Secretion, Dr. George W. Crile of Cleveland; February 13, (subject to be announced) Dr. Irving M. Snow; February 20, Uterine Displacements, Dr. Charles W. Banta; Prevention of Malpositions of the Uterus and Allied Disorders, Dr. John V. Woodruff;

February 27, (subject to be announced) Dr. Wm. H. Welch, Baltimore.

---

THE annual meeting of the Oneida County Medical Society was held at Utica, January 9, 1912, Dr. Fayette H. Peck presiding. The scientific program was as follows:

"Immediate repair of soft parts after labor," Dr. Angeline Martine; "Puerperal eclampsia," Dr. W. J. Schuyler; "Indications for Forceps," Dr. F. D. Crim; "Preparation and Care of Patients in an Ordinary Accouchement," Dr. M. J. Davies; "Relations of Pregnancy to Certain Abdominal Diseases," Dr. A. L. Benedict, Buffalo.

Officers elected: President, Dr. T. H. Farrell; Vice-President, C. R. Hart; Secretary, W. B. Roemer; Treasurer, T. Wood Clark; Librarian, Smith Baker; Censors, F. J. Douglass, H. G. Jones, Charles Bernstein, E. D. Fuller, F. D. Crim; Delegates to State Society, F. H. Peck; alternates, Charles H. Baldwin, D. M. Allison, H. G. Jones; Delegates to 5th District Branch, T. Z. Jones, T. Wood Clark; alternates, G. J. Ballard, Kent E. Williams.

---

THE Medical Union of Buffalo gave a banquet to nearly a hundred members and guests, at the Hotel Statler, January 24, 1912, in honor of Major Frederick F. Russell, M. D., U. S. A. The President, Dr. George F. Cott, called upon Major Wm. G. Bissell to introduce the guest of the evening, and the latter read a scholarly paper on the use of Typhoid Vaccine, which was briefly discussed by Drs. H. C. Buswell and James E. King. Many of those present expressed the wish that we might publish the paper in full and we would gladly have done so but, as the material was based on official records, Major Russell did not feel at liberty to publish it at present. We may, however, state that, largely due to the work of our own army medical staff, typhoid vaccine has been made much more efficient and freer from danger than during the Boer war; that it has been employed in about one hundred thousand Americans, under the direction of the U. S. A. surgeons without any serious and with few unpleasant immediate results; that immunization for about two and a half years is apparently produced by the use of three doses in about three weeks; that the incidence in vaccinated cases, apparently subjected to risk of infection, is much less than for the unprotected. Major Russell also made a point which few realize: that army manoeuvres may be shams so far as the strictly military features are concerned but that, for the medical staff, they lack only traumatic and emergency features to afford real experience.

The Elmira Academy of Medicine will meet, February 7, 1912, at the Federation Building. Dr. John C. Fisher will present a paper on Neurasthenia. Non-members are invited.

---

The Medical Society of the County of Allegany met at the Kinney House, Cuba, January 11, 1912. Papers were read by Dr. N. H. Fuller of Friendship on Scarlet Fever and by Dr. F. E. Brundage of Belmont, on Children.

---

The annual meeting of the Rochester Academy of Medicine was held at Hotel Seneca, January 17th, 32 fellows being present.

The proposed amendment to the By-Laws, "That each section shall meet once in four months or oftener if it is so desired, except during the months of June, July, August and September, which shall be deemed vacation months," was adopted.

A proposed amendment to limit the membership of the Academy, was tabled indefinitely.

Officers were elected as follows:

President, Charles E. Darrow, M. D.; Vice-presidents, in charge of the work of the four sections, Cornelia White-Thomas, M. D., Charles E. Van Der Beek, M. D., William W. Percy, M. D., Leonard W. Jones, M. D.; Treasurer, Wesley T. Mulligan, M. D.; Secretary, Edward L. Hanes, M.D., appointed by the Council to fill the unexpired term of William M. Brown, M. D., resigned. Trustees, Loren W. Howk, M. D., Richard M. Moore, M. D., and Thomas A. O'Hare, M. D.; Councillors, William B. Jones, M. D., and Charles D. Young, M. D.; Member of Library Committee, John O. Roe, M. D.

The retiring President, Dr. Richard M. Moore, presented his address in the form of a most interesting and suggestive paper on the question of keeping tuberculous patients at home under the care of the family physician.

Following the adjournment the newly elected President, Dr. Charles E. Darrow, appointed a new committee to take up the much discussed question of a home for the Academy:

Dr. Edward B. Angell, Chairman,
Dr. Edward Mulligan,
Dr. Willis E. Bowen.

The names of eight candidates for election to fellowship were presented.

